# Evaluation of a subunit vaccine candidate (Biotech Vac Cox) against *Eimeria* spp. in broiler chickens

**DOI:** 10.1016/j.psj.2021.101329

**Published:** 2021-06-11

**Authors:** Emanuel Gumina, Jeffrey W. Hall, Bruno Vecchi, Xochitl Hernandez-Velasco, Brett Lumpkins, Greg Mathis, Sherry Layton

**Affiliations:** ⁎ Vetanco SA, Villa Martelli B1603, Province of Buenos Aires, Argentina; † Vetanco USA, Saint Paul, MN 55114, USA; ‡ Department of Avian Medicine and Zootechnics, FMVZ, National Autonomous University of Mexico, Mexico City 04510, Mexico; § Southern Poultry Research, Athens, GA 30607, USA; # BV Science, Lenexa, KS 66219, USA

**Keywords:** apicomplexa, broiler, coccidiosis, *Eimeria*, subunit vaccine

## Abstract

This study evaluated growth performance and cross-protection against *Eimeria* spp*.* using a subunit coccidia vaccine in 2 independent challenge experiments. In both trials, chickens were challenged with *E. acervulina, E. maxima*, and *E. tenella* oocysts. In Exp 1, 1000-day-old chickens were allocated in one of 2 treatments 1) Control group; 2) Biotech Vac Cox group. The vaccine was orally gavaged on d 2 and 16 of life and coccidia challenge was on d 21. Performance parameters were evaluated on d 21, 35, and 42. On d 34, coccidia lesions were scored. Oocysts per gram of feces (**OPG**) were evaluated on d 28, 35, and 42. In Exp 2, 900-day-old chickens were assigned in one of 2 treatments 1) Control group; 2) Biotech Vac Cox group. The vaccine was orally gavaged on d 2 and 16 of life and coccidia challenge was on d 21. Performance parameters were evaluated on d 21, 27, 35, and 42, and lesion scores and OPG at d 27. In Exp 1, chickens vaccinated had significantly lower feed intake (**FI**) at d 21 and feed conversion ratio (**FCR**) at d 35 compared to control chickens (*P* < 0.05). Vaccinated chickens showed a significant reduction (*P* ≤ 0.05) in OPG for *E. maxima* to nondetectable levels and for all coccidian species at d 42 compared to control chickens. In Exp 2, the chickens vaccinated showed a significant increase in BW, BW gain (**BWG**) and reduction in FCR on d 27, 35, and 42 (*P* ≤ 0.05). Vaccinated chickens had significantly lower (*P* ≤ 0.05) lesion scores for all 3 *Eimeria* species. Moreover, vaccinated chickens had a reduction in total OPG of 35.50% (*P* = 0.0739). Studies to evaluate the serological and mucosal immune response are currently being evaluated. This inactivated, orally delivered subunit vaccine offers significant cross-protection to *Eimeria* spp*.* and eliminates the needs to treat broilers with live oocysts, enhanced ease of use, and greater biosecurity to producers.

## INTRODUCTION

The apicomplexan phylum of protozoa, characterized by the presence of an “apical complex”, contains numerous parasites of veterinary (*Cryptosporidium, Neospora, Eimeria*) and medical (*Plasmodium, Cryptosporidium, Toxoplasma*) importance (Sato, 2011). *Eimeria* spp. are the causative agent of coccidiosis, which continues to be one of the most critical enteric diseases in the commercial poultry industry, with losses to the sector estimated to be 2 billion euros (Peek and Landman, 2011). Coccidiosis manifests in the gastrointestinal tract (**GIT**), resulting in severe diarrhea and affecting growth performance with subsequent increases in feed conversion ratio and mortality (Hernández-Velasco et al., 2014). The life cycle of *Eimeria* is complex and involves both intracellular and extracellular stages (Yun et al., 2000). Each *Eimeria* spp.*,* colonizes specific areas of the GIT depending on its specificity (Lillehoj and Trout, 1996).

Conventional disease control approaches have employed prophylactic medications, such as chemotherapy and anticoccidials agents, and the selection of disease-resistant strains of chickens (Chapman, 1997). However, with the ability of parasites to develop drug resistance, research into alternative methods of disease prevention and control continues (Chapman, 1999; Allen and Fetterer, 2002). In general, *Eimeria* spp. is potently immunogenic and can elicit a strong cellular immune response (Williams, 2002). In this regard, vaccination against coccidiosis has become an essential aspect of commercial poultry production and continues to be actively researched (Lillehoj and Trout, 1993; Song et al., 2000; Dalloul and Lillehoj, 2006). However, current commercial vaccines are hindered by their complex production processes and species-specific protection (Peek and Landman, 2011). An ideal vaccine candidate should stimulate a significant cross-species immune response and confer long-term protection (Lillehoj and Trout, 1993).

Immunity to the disease is complex and involves many facets of the host immune system (Chase, 2018). There is a definite interplay between humoral and cell-mediated immunity, though it is accepted that cell-mediated immunity is more important (Lillehoj and Trout, 1996; Hong et al., 2006a). The parasite is known to colonize the intestinal epithelium. Hence, the primary line of host defense is mucosal-associated lymphoid tissue (Schnitzler and Shirley, 1999; Shirley et al., 2007). The mucous membranes constitute the primary portal of entry for infectious agents. They include membranes of the respiratory, gastrointestinal, and genitourinary tract, the ocular conjunctiva, the inner ear, and the ducts of all exocrine glands (Ogra et al., 2001). Collectively they cover more than 400 m^2^ in humans and serve as the first line of defense against infection at the entry points for various pathogens (Ogra et al., 2001). It is estimated that the gut-associated lymphoid tissue (**GALT**) comprises over 70% of the body's total immune cells and about 80% of all plasma cells, which are primarily IgA-producing cells (Vighi et al., 2008). The concept of a standard mucosal immune system predicts that induction of immunity at one mucosal surface, such as the gut, can provide immunity at another mucosal surface, such as the lung providing a vital link for immunity transfer throughout mucosal surfaces (Turner, 2009).

The objective of this research was 3-fold: 1) identify an immunologically protective pan-apicomplexan antigen, 2) develop an orally amendable delivery system, and 3) evaluate the cross-protection against 3 *Eimeria* species induced by this vaccine in broiler chickens.

When selecting the protective protein or subunit for inclusion in our universal vaccine against apicomplexan parasites, 4 criteria were considered. 1) The protein sequence should be highly conserved. More specifically, the protein sequence should be identical for all species and serotypes or strains of the species; 2) the protein must be accessible to the immune system on the pathogen; 3) the protein should be antigenic and immunogenic when presented alone in a recognizable fashion to the host; 4) the immune response (humoral or cell-mediated but preferably both) should be relatively quick, efficient, protective, and long-lasting. The antigen selected for the vaccine candidate tested is comprised a highly conserved hypothetical protein with the *Eimeria* genera that is predicted to be a part of the microneme. Proteolytic trimming of microneme contents occurs rapidly after their secretion onto the parasite surface and is proposed to regulate complex adhesive activation to enhance binding to host cell receptors (Dowse and Soldati, 2004). Microneme proteins are also critical for the motility of the protozoa as it moves toward the host cell (Keeley and Soldati, 2004). It has been demonstrated that protozoa, which lack these proteins, have a profound defect in the surface processing of secreted microneme proteins (Lagal et al., 2010). Notably, parasites lack protease activity responsible for proteolytic trimming of MIC2, MIC4, and M2AP after release onto the parasite surface (Dalloul and Lillehoj, 2005). Loss of this proteolytic protein decreases cell attachment and in vitro gliding efficiency leading to lower invasion rates. Since protozoa must invade host cells to carry out their replication, lower rates of invasion negatively affect replication (Cornelissen and Schetters, 1996). Thus, impacting the number of protozoa available to cause disease and be shed back into the environment. If this protein is disrupted by an immune response within the host species, the protozoa are less likely to invade host enterocytes, less likely to replicate, and less likely to be able to cause disease; making this protein an excellent target for vaccination purposes (Allen and Fetterer, 2002). Several recent publications have described targeting *E. tenella* specific microneme proteins further validating the Apicomplexa microneme organelle as a valid vaccine target (Yan et al., 2018; Wang et al., 2020; Chen et al., 2021; Song et al., 2021).

Increasing evidence has indicated that mucosal vaccination can induce systemic and local mucosal immunity, while systemic immunization generally fails to elicit solid mucosal immunity (Strober et al., 2002; Bienenstock and McDermott, 2005). Vaccines that are administered through a mucosal route of entry that can elicit mucosal, humoral, and cell-mediated immune responses offer a promising alternative approach when compared with existing traditional (inactivated subcutaneous or orally administered attenuated total pathogen) vaccine strategies (Jayawardane and Spradbrow, 1995; Johansen and Brandtzaeg, 2004). In this regard, the field of vaccinology has recently undergone a transformation from a more traditional belief that systemic immunity is the only effective way to generate protection against infectious diseases to a more progressive thought process of effective immunity that can be achieved through mucosal immunity (Cornelissen and Schetters, 1996). Despite its important role, only a handful of vaccines specifically target this area of the immune system despite strong evidence that a robust mucosal response can effectively prevent systemic infections (Stevceva et al., 2000). The use of recombinant vectored vaccines for disease control and protection has been well documented (Crane et al., 1991; Allen and Fetterer, 2002). Simple approaches to design and construction have been evaluated and used successfully in experimental models. A large number of parasite antigens have been employed as vaccine candidates to confer protection (Song et al., 2000). Several vaccine vectors have emerged to date, all of which have relative advantages and limitations depending on the proposed application (Yun et al., 2000). However, bacterial vectors have been regarded as the front runner in vectored vaccine strategies. Both *Lactobacillus* spp. and most *Bacillus* spp. are considered generally recognized as safe or GRAS organisms with a very comprehensive record of safe oral consumption, widely known for their use in food fermentation processes as probiotics (Amuguni and Tzipori, 2012). Most of our current knowledge surrounding the use of both *Lactobacillus* and *Bacillus* as a vectored vaccine strategy centers around the use of the Tetanus Toxin Fragment C (**TTFC**) as a fusion protein integrated within the chromosome or on a stable plasmid (Norton et al., 1997; Reveneau et al., 2002; Cortes-Perez et al., 2007). *Bacillus* bacteria, specifically *Bacillus subtilis*, provide a promising alternative to the use of pathogenic bacteria as a live oral vectored vaccine (Hoang et al., 2008). Furthermore, *Bacillus* possesses inherent adjuvant activity potentiating stimulation of host-specific immunity through the toll-like receptor pathways, fortify the gastrointestinal system by enhancing the production of tight junction repair proteins and down-regulate the inflammatory response caused by pathogenic Gram-negative bacteria (Hong et al., 2006b; Cartman et al., 2007). These properties combined make *Bacillus* a very attractive candidate for use as oral recombinant vaccines (Huang et al., 2010), as they have been shown to stimulate systemic, mucosal, innate, humoral, and cell-mediated immune responses against heterologous antigens (Cornelissen and Schetters, 1996; Yu and Cutting, 2009), providing the possibility of greatly enhanced protection compared to parenteral vaccination (Yurong et al., 2005; Amuguni and Tzipori, 2012).

A primary impediment to developing an immunological protective orally administered vaccine is the harsh environmental conditions of the gastrointestinal tract that promote the degradation of the antigen before the immune system can recognize the antigen, respond accordingly, and provide protection against the intended target pathogen (Martin et al., 1997). This problem has been overcome by using a novel naturally occurring polysaccharide carrier that protects the protective protein and probiotic properties of the bacilli bacteria.

## MATERIAL AND METHODS

### Subunit Vaccine Candidate Construction

Biotech Vac Cox (Vetanco S.A. Buenos Aires, Argentina) was created by transforming *Bacillus subtilis* with a proprietary *Bacillus* expression plasmid; this plasmid is responsible for the production and transportation of the subunit, or protective protein, to the cell membrane of the *Bacillus subtilis*. Production of the subunit is controlled by a classical LacI/P*spac* repressor and promoter cassette and transcription can be induced with isopropyl β-D-1-thiogalactopyranoside (IPTG).

The protective protein was first characterized in *Toxoplasma gondii* (Baum et al., 2008; Lagal et al., 2010). Since *Toxoplasma* and *Eimeria* are phylogenetically similar, the *T. gondii* protein sequence was entered into the National Center for Biotechnology Information (**NCBI**) BLASTP server to identify the orthologous protein in *Eimeria* spp. Once the protein sequence was identified, a gene sequence was derived in silico using codon optimization parameters for *B. subtilis,* and a gene was synthesized (GenScript). This synthetic gene was amplified using traditional PCR with gene-specific primers. The 5’ ends of the forward and reverse oligonucleotides contained restriction endonuclease sequences, *BamH*I and *Xba*I, respectively. The amplification product was purified by gel extraction techniques, concentrated, digested with *BamH*I and *Xba*I, and repurified. A proprietary expression plasmid was digested with *BamH*I and *Xba*I, purified, concentrated, and treated with rSAP. The gene insert and plasmid were then mixed into a T4 DNA ligase reaction overnight at RT. The ligation reaction was transformed into *E. coli* DH5α (Invitrogen) and transformants were screened for the gene insert on LB agar with ampicillin (100 µg/mL, LB^Amp^). The new plasmid, pCox, was purified from *E. coli,* and transformed into *B. subtilis*. Transformants were selected on tryptic soy agar with chloramphenicol (5 µg/mL, TSA^Cm^), creating Biotech Vac Cox (Blake et al., 2006).

### Vaccine Production

The protective subunit is produced by inoculating a fermenter with a 1:10 dilution of an overnight culture of the recombinant *Bacillus*. The fermentation proceeds for 3 h at 37°C at which time antigen production is induced with 0.5 m*M* IPTG for 8 h. The culture is then inactivated with formalin (0.1% v/v). To produce the complete vaccine, Biotech Vac Cox, the inactivated culture was mixed and with a proprietary acidic encapsulation media containing methylcellulose and stirred together overnight. The methylcellulose solution microencapsulates the bacteria and antigen and acts as the vehicle to protect the antigen as it passes through the gastrointestinal tract (Rogers and Wallick, 2011; Wallick, 2014).

### Facilities

All experiments of this study were conducted at Southern Poultry Feed and Research facilities (**SPFR**), Athens, Georgia. The experimental house has equal size pens, each having an area of 3.72 m^2^, with fresh wood shavings as bedding with a thickness of approximately 10 cm on a dirt floor. Each pen has 1.5-m-high sidewalls with a 0.5-m bottom of solid wood to prevent bird migration. The temperature of the building was monitored daily and was optimum for the age of the birds according to the breed requirements. Illumination was provided by fluorescent bulbs placed above the pens. The experiment was conducted in accordance with the principles and specific guidelines presented in the Federation of Animal Science Societies (FASS, 2020).

### Experimental Animals

The birds were obtained from a Cobb-Vantress hatchery, Cleveland, Georgia. Bird sexing was done at the hatchery without administration of any coccidia vaccine.

### Trial Diets and Water Supply

Diets for all experiments were manufactured at SPFR Feed Mill. All feeds were fed as crumbles/pellets. All diets did not contain any anticoccidial drug. However, all feeds contained Bacitracin Methylene Disalicylate (**BMD**) 11% at 0.5 kg/metric ton. In all experiments, all feed was weighed by pen. Starter feed was fed from d 0 to 21. On d 21, the nonconsumed Starter was weighed and discarded. Grower feed was issued and fed until d 35. On d 35, the nonconsumed Grower was weighed and discarded. Finisher feed was issued and fed until d 42. On d 42, the nonconsumed Finisher was weighed and discarded. Diet composition was appropriate for the birds' growing stage (Table 1) and met the NRC (1994) requirements. Water was provided *ad libitum* from one Plasson-type automatic watering fount per pen. Performance parameters in all experiments included BW, BWG, FI, and FCR.

**Table 1:** Ingredient composition and nutrient content of the corn-soybean diet used on as-is basis.

Ingredient	Starter %0–21 d	Grower %22–35 d	Finisher %36–42 d
Corn (%)	57.107	63.672	69.218
Soybean meal (%)	36.859	30.579	24.511
Fat, vegetable (%)	2.337	2.444	2.748
Dicalcium phosphate (%)	1.407	1.263	1.693
Calcium carbonate (%)	1.26	1.051	0.873
Salt (%)	0.437	0.441	0.393
DL-methionine (%)	0.315	0.235	0.260
L - lysine (%)	0.096	0.172	0.180
Vitamin premix^1^ (%)	0.075	0.075	0.075
Trace mineral premix^2^ (%)	0.065	0.050	0.050
L-threonine 98.5 (%)	0.023	0.000	0.000
Phytase^3^ (%)	0.019	0.019	0.019
Calculate analysis			
Crude protein (%)	22.5	20	17.2
ME, kcal/kg	3,054	3,130	3,180
Digestible methionine (%)	0.60	0.50	0.49
Digestible cysteine (%)	0.30	0.28	0.25
Digestible lysine (%)	1.20	1.1	0.95

1 Vitamin mix provided the following (per kg of diet): 2.4 mg of thiamine mononitrate; 44 mg of nicotinic acid; 4.4 mg of riboflavin; 12 mg of d-Ca pantothenate; 12.0 µg of vitamin B12 (cobalamin); 4.7 mg of pyridoxine·HCl; 0.11 mg of d-biotin; 5.5 mg of folic acid; 3.34 mg of menadione sodium bisulfite complex; 220 mg of choline chloride; 27.5 µg of cholecalciferol; 6,306.6 IU of trans-retinyl acetate; 11 IU of all-rac α-tocopheryl acetate; 125 mg of ethoxyquin.

2 Trace mineral mix provides the following (per kg of diet): 60 mg of manganese (MnSO_4_·H_2_O); 30 mg of iron (FeSO_4_·7H_2_O); 50 mg of zinc (ZnO); 5 mg of copper (CuSO_4_·5H_2_O); 0.15 mg of iodine (ethylene diamine dihydroiodide); 0.3 mg of selenium (NaSeO_3_).

3 Ronozyme P-(CT) (DSM Nutritional Products, Ames, IA).

### Coccidia Species Challenge

In Experiments 1 and 2, chickens were challenged with approximately 100,000; 50,000; and 75,000 *Eimeria acervulina, Eimeria maxima*, and *Eimeria tenella* oocysts, respectively. All coccidia used were contemporaneous and isolated from U.S.A. commercial production farms. For each species, the dose was selected to produce a moderate coccidiosis infection. The oocysts were mixed, giving a total volume of 550 mL. Each inoculum had 30 mL and was mixed into the feed found at the base of the tube feeder of the challenged pens, where each pen had 50 birds.

### Trial Monitoring

Birds and housing facilities were inspected twice daily. During the inspections, the following were checked and recorded: general health status, constant feed and water supply, temperature, removal of all dead birds, and observation of any unexpected events. The number of birds found dead during the study was noted on the daily mortality record, and the birds were not replaced.

### Coccidiosis Lesion Scoring

Following euthanasia by cervical dislocation, 5 representative chickens (per pen) in both trials were examined for the presence and degree of coccidiosis lesions. The upper, middle, and cecal regions of the intestinal tract were scored, using the system of Johnson and Reid (1970), where 0 is normal, and 1, 2, 3, or 4 indicate increasing severity of the infection.

### Oocyst Counting

In each experiment, fresh fecal samples were collected per pen, pooled, and kept in separate airtight plastic bags. After homogenization, samples were stored at 4°C until assessed for oocyst counts, determined by dilution, and counts via microscope using a McMaster counting chamber and stated as oocysts per gram of feces (**OPG**) (Haug et al., 2006).

### Disposal of Birds and Feed

All birds and feed were buried in the Southern Poultry Research's pit as described in the Southern Poultry Feed and Research standard operating procedure.

### Experimental Design: Experiment 1

One-thousand-day-old male Cobb 500 chickens were allocated in one of 2 treatments 1) Challenged, control group; 2) Challenged, Biotech Vac Cox group. The treatments were replicated in ten blocks, randomized within blocks of 2 pens each, and n = 50 broiler chickens/replicate. The vaccine was gavaged (0.2 mL/bird) individually on d 2 and 16. Performance parameters were evaluated on d 21, 35, and 42. On d 21, all chickens were challenged with a combination of *E. acervulina, E. maxima,* and *E. tenella*. Six days postchallenge (d 34), 5 representative birds were removed from each pen (n = 50), tagged with pen number, group weighed. These birds were euthanized, and coccidia lesions scored. On d 28, 35, and 42 fresh fecal samples were collected from each pen. These representative samples were analyzed to determine the degree of oocysts shedding/cycling.

### Experimental Design: Experiment 2

Nine-hundred-day-old male Cobb-Vantress chickens were allocated in one of 2 treatments 1) Challenged, control group; 2) Challenged, Biotech Vac Cox group. The treatments were replicated in 9 pens, randomized within blocks of 2 pens each, and n=50 broiler chickens/replicate. The vaccine was gavaged (0.2 mL/bird) individually on d 2 and 16. Performance parameters were evaluated on d 21, 27, 35, and 42. On d 21 all chickens were challenged with coccidia. Six days postchallenge (d 27), 5 representative birds were removed from each pen (n = 45), tagged with pen number and group weighed. These birds were euthanized, and coccidia lesions scored. On d 27, fresh fecal samples were collected from each pen. These representative samples were analyzed to determine the degree of oocysts shedding/cycling.

### Statistical Analysis

All data were subjected to ANOVA as a completely randomized design using the GLM procedure of SAS (SAS Institute, 2002). For evaluation of growth performance parameters (BW, BWG, FI, and FCR), each of the replicate pens was considered as the experimental unit in each experiment, respectively. For evaluation of lesion scores, each chicken was considered as an experimental unit. Treatment means were partitioned using Duncan's multiple range test at α ≤ 0.05, indicating statistical significance.

## RESULTS

### Experiment 1

The results of the evaluation of Biotech Vac Cox on broiler chickens on performance parameters in Experiment 1 are summarized in Table 2. No significant differences were observed between control or vaccinated chickens on BW or BWG at 21, 35, or 42 d of evaluation (*P* > 0.05). In this experiment, the only significant changes were observed on FI at d 21 and FCR at d 35 in vaccinated chickens compared to control chickens. No significant changes were observed in mortality between control and vaccinated chickens (Table 2).

**Table 2:** Evaluation of Biotech Vac Cox on broiler chickens on performance parameters in Experiment 1.

	No vaccine control	Biotech Vac Cox	Pooled SEM	*P-*value
**BW, kg/broiler**				
d 21	0.59	0.57	0.03	0.1900
d 35	1.62	1.61	0.08	0.0600
d 42	2.15	2.17	0.06	0.5100
**BWG, kg/broiler**				
d 21	0.55	0.53	0.03	0.2000
d 35	1.58	1.57	0.04	0.9600
d 42	2.11	2.13	0.06	0.5100
**FI, kg/broiler**				
d 21	0.89^a^	0.84^b^	0.03	0.0008
d 35	2.97	2.89	0.089	0.0660
d 42	4.09	4.04	0.11	0.3900
**FCR**				
d 21	1.48	1.46	0.05	0.2300
d 35	1.66^a^	1.62^b^	0.03	0.0200
d 42	1.73	1.70	0.04	0.1200
**Total mortality**	47/500 (9.4 %)	49/500 (9.8 %)		

ab Indicates significant differences between the treatments within the rows (*P* ≤ 0.05) from 10 replicates/treatment and n = 50 chickens/replicate.

Table 3 shows the results of the evaluation of Biotech Vac Cox on broiler chickens challenge with coccidia on lesion scores and OPG in Experiment 1. Lesion scores on d 34 (Johnson and Reid, 1970) were assessed as a measure of coccidia-induced damage (*E. acervulina, E. maxima, E. tenella*, and total average) to the gastrointestinal tract. Chickens vaccinated with Biotech Vac Cox had significantly lower lesion scores for all 3 *Eimeria* species and in total average lesion scores when compared to the nontreated controls. Average lesion scores were reduced by 57.53%. The percent reduction in lesion scores for the individual *Eimeria* species between Biotech Vac Cox and nontreated control groups were as follows: *E. acervulina* 64.95%, *E. maxima* 57.89%, and *E. tenella* 40% (Table 3). At d 28, no significant differences were observed in OPG between treated or control chickens. However, at d 35, vaccinated chickens showed a significant reduction in OPG for *E. maxima* to nondetectable levels. Promisingly, on d 42, a significant reduction in OPG was observed for all coccidian species in vaccinated chickens than control chickens. These data suggest that the protozoa are not replicating and are simply transient (Table 3).

**Table 3:** Evaluation of Biotech Vac Cox on broiler chickens challenge with coccidia on lesion scores and oocyst per gram of feces (OPG) in Experiment 1.

	Control	Biotech Vac Cox	Pooled SEM	*P-*value
**Lesion scores (d 34)**				
*E. acervulina*	2.34^a^	1.52^b^	0.25	0.0001
*E. maxima*	1.14^a^	0.66^b^	0.24	0.0003
*E. tenella*	0.90^a^	0.36^b^	0.33	0.0002
Average	1.46^a^	0.84^b^	0.16	0.0001
**OPG (d 28)**				
*E. acervulina*	2,891	1,714	2,139	0.2345
*E. maxima*	1,037	333	1,003	0.1342
*E. tenella*	220	366	290.95	0.2740
Total	4,149	2,415	2,904	0.1986
**OPG (d 35)**				
*E. acervulina*	593	240	387	0.0564
*E. maxima*	313^a^	0.00^b^	154	0.0003
*E. tenella*	987	86	1,931	0.3110
Total	1,894	326	1,813	0.0691
**OPG (d 42)**				
*E. acervulina*	360^a^	66^b^	201	0.0044
*E. maxima*	153^a^	0.00^b^	80	0.0005
*E. tenella*	93^a^	40^b^	51	0.0328
Total	607^a^	106^b^	275	0.0007

On d 21, all chickens were challenged with a combination of *E. acervulina, E. maxima,* and *E. tenella*. Six days postchallenge (d 34) 5 representative birds were removed from each pen (n = 50) and were euthanized for coccidia lesion scored infection.

ab Indicates significant differences between the treatments within the rows (*P* ≤ 0.05).

### Experiment 2

The results of the evaluation of Biotech Vac Cox on broiler chickens on performance parameters in Experiment 2 are summarized in Table 4. In this experiment, chickens that received the vaccine showed a significant increase in BW, BWG and significant reduction in FCR on d 27, 35, and 42. Nevertheless, no significant differences were observed in FI or in mortality between both experimental groups (Table 4).

**Table 4:** Evaluation of Biotech Vac Cox on broiler chickens on performance parameters in Experiment 2.

	No vaccine control	Biotech Vac Cox	Pooled SEM	*P-*value
**BW, kg/broiler**				
d 21	0.72	0.70	0.05	0.4420
d 27	0.972^b^	1.04^a^	0.03	0.0038
d 35	1.46^b^	1.58^a^	0.06	0.0147
d 42	1.98^b^	2.14^a^	0.12	0.0153
**BWG, kg/broiler**				
d 21	0.68	0.66	0.05	0.4387
d 27	0.94^b^	1.02^a^	0.05	0.0077
d 35	1.42^b^	1.54^a^	0.09	0.0146
d 42	1.94^b^	2.10^a^	0.12	0.0153
**FI, kg/broiler**				
d 21	0.99	0.96	0.05	0.3668
d 27	1.74	1.71	0.04	0.2552
d 35	3.17	3.14	0.10	0.5842
D 42	4.55	4.56	0.14	0.9996
**FCR**				
d 21	1.36	1.37	0.04	0.9791
d 27	1.76^a^	1.60^b^	0.03	0.0200
d 35	1.98^a^	1.82^b^	0.06	0.0001
d 42	2.13^a^	1.98^b^	0.05	0.0001
**Total mortality**	15/450 (3.33 %)	18/450 (4.00 %)		

ab Indicates significant differences between the treatments within the rows (*P* ≤ 0.05) from 9 replicates and n = 50 chickens/replicate.

Table 5 shows the results of the evaluation of Biotech Vac Cox on broiler chickens challenged with coccidia on lesion scores and OPG in Experiment 2. Chickens vaccinated with the Test Vaccine had significantly lower lesion scores for all 3 *Eimeria* species and in total average lesion scores when compared to the nontreated controls. Average lesion scores were reduced by 55.27% in the Biotech Vac Cox group as compared to the nontreated controls; percent reduction in lesion scores for the individual *Eimeria* species between the Biotech Vac Cox group and nontreated control groups were as follows: *E. acervulina* 61.88%, *E. maxima* 61.86%, and *E. tenella* 33.57%. Although in this experiment, no differences were observed in OPG at d 27 between control and vaccinated chickens, vaccinated chickens tended to show a reduction in total OPG of 35.50% (*P* = 0.0739) that was reflected by numerical reductions in OPG for all 3 *Eimeria* species evaluated (Table 5).

**Table 5:** Evaluation of Biotech Vac Cox on broiler chickens challenge with coccidia on lesion scores and oocyst per gram of feces (OPG) in Experiment 2.

	Control	Biotech Vac Cox	Pooled SEM	*P-*value
**Lesion scores (d 27)**				
*E. acervulina*	2.44^a^	1.51^b^	0.39	0.0001
*E. maxima*	2.15^a^	1.33^b^	0.51	0.0040
*E. tenella*	1.37^a^	0.46^b^	0.52	0.0019
Average	1.99^a^	1.10^b^	0.43	0.0005
**OPG (d 27)**				
*E. acervulina*	13,817	3,447	11,285	0.0690
*E. maxima*	2,859	454	3,321	0.1441
*E. tenella*	8,174	4,921	8,370	0.4218
Total	24,850	8,822	17,779	0.0739

On d 21, all chickens were challenged with a combination of *E. acervulina, E. maxima,* and *E. tenella*. Six days postchallenge (d 27), 5 representative birds were removed from each pen (n = 45) and were euthanized for coccidia lesion scored infection.

ab Indicates significant differences between the treatments within the rows (*P* ≤ 0.05).

## DISCUSSION

Coccidiosis in poultry is a common, reoccurring disease, which has global importance in the commercial industry. Effective vaccination against coccidiosis has become one of the most sought-after aspects of modern-day poultry research and is considered a viable option for disease control (Dalloul and Lillehoj, 2006). In the present study, an inactive, oral subunit vaccine candidate was tested against a direct *E. acervulina, E. maxima*, and *E. tenella* challenge. The candidate subunit vaccine tested in the present study was able to significantly reduce the severity of the lesions of all 3 coccidian species evaluated in 2 independent experiments. In Exp 1, no detection of OPG for *E. maxima* was observed at 35 and 42 d of evaluation. A significant reduction in OPG for *E. acervulina* and *E. tenella* was observed at 42 d of evaluation. These data suggest that the protozoa are not replicating and are simply transient. Furthermore, in Exp 2, the subunit vaccine was able to increase the performance parameters significantly, and in this trial, although not significant, there was a tendency to reduce OPG for all strains. In addition to the growth performance gains, the reduction in OPG of feces reduces environmental contamination with oocysts, thereby reducing the pathogenic load on the next production cycle when litter is reused. Continual and consistent use of this novel vaccine may lead to a significant and steady reduction in the use of anticoccidials and disease.

Current production methods of live-attenuated coccidia vaccines are cost prohibitive and induce only species-specific immunity. Recent advances in immuno-proteomics have identified immunodominant *Eimeria* antigens, which has led to the development of novel vaccines to combat this disease; unfortunately, most of these vaccines have focused on *E. tenella* and either do not confer, or lack, experimental evaluated immunity to *E. acervulina* and *E. maxima*.

Here, we present evidence that an inactivated, orally administered, subunit comprised of a highly conserved apicomplexan antigen can effectively protect broiler chickens from coccidiosis by direct challenge with *E. tenella, E. acervulina,* and *E. maxima*. This vaccine can be easily administered in the water supply system and is commercially scalable and cost-effective to produce.

Studies to evaluate the serological and mucosal immune response are currently being evaluated. These data indicate a single vaccine, Biotech Vac Cox, can be developed to effectively vaccinate against at least 3 economically important and pathogenic *Eimeria* species.
